# The pathogenesis of post-stroke osteoporosis and the role oxidative stress plays in its development

**DOI:** 10.3389/fmed.2023.1256978

**Published:** 2023-10-19

**Authors:** JinYan Li, Lin Shi, JianMin Sun

**Affiliations:** ^1^School of Clinical Medicine, Weifang Medical University, Weifang, China; ^2^Weifang People’s Hospital, Weifang, China

**Keywords:** cardiovascular disease, stroke, bone mineral density, osteoporosis, oxidative stress, vitamin D

## Abstract

Cardiovascular disease and osteoporotic fractures (OF) are the main diseases affecting the health of middle-aged and elderly people. With the gradual increase of population aging in China and even the world, the incidence of the two and the prevalence of high-risk groups are also showing a continuous upward trend. The relationship between the two, especially the impact of cardiovascular disease on the risk and prognosis of OF, has attracted more and more attention. Therefore, it is of great significance to fully understand the pathogenesis of cardiovascular and cerebrovascular diseases and the resulting osteoporosis and to provide targeted interventions to prevent the occurrence of diseases and fractures. This article reviews the relationship between one of the Cardiovascular disease—stroke and related therapeutic drugs and the risk of OF, and the role of oxidative stress in its pathophysiological mechanism by reviewing relevant domestic and foreign literature in recent years, in order to gain a more comprehensive understanding of the association between stroke and OF, and then provide a basis and reference for screening high-risk groups of fractures and reducing the burden on the health system caused by the disease.

## Highlights

– The following criteria are used to determine the appropriate study to study the relationship between stroke and osteoporosis;
– A comparative study including stroke patients and healthy people;
– To provide the information of bone mineral density (BMD) level in case group and control group at the onset of osteoporosis after stroke;
– Study on the relationship between oxidative stress and stroke and osteoporosis;
– Research on the present situation and prospect of osteoporosis treatment after stroke;
– Study on evaluating the correlation between stroke and osteoporosis;
– Case-control study in Chinese and English;
– Chinese and English documents on case reports, meeting minutes, abstracts, communications and news developments;
– Research on maxillofacial osteoporosis;
– The data of the conclusion is incomplete;
– documents that are too old (if necessary).

## 1. Introduction

Stroke is the second leading cause of death in the world (1), and it is a major health problem that seriously harms human health. With the growth of patients’ age, the death rate of stroke will increase rapidly. As shown in Table 1, Zhang (2) gave the incidence of osteoporosis in patients with and without stroke. The risk of fracture, disability and death is higher in elderly stroke patients. The study found that the fracture risk of stroke population is four times higher than that of the non-stroke population. According to statistics, 3–6% of stroke patients have fractures within 1 year after stroke (2). In addition, the risk of fractures after stroke also exists in other ethnic groups. Lisabeth (3) et al., found that, among non-Hispanic white people and Mexican Americans, a 3% increased risk of fracture at 1 year and a 10% increased risk at 5 years after stroke were found. Benzinger et al. (4), found in a German cohort study that the risk of OF after stroke was higher, the fracture incidence density of non-stroke patients was 21.4/1000 person-years, and the fracture incidence density of stroke patients was 33.6/1000 person-years. It can be seen that cerebrovascular disease (stroke) is closely related to OF, and the risk of OF may be further increased after stroke.

**TABLE 1:** Incidence and HRs of osteoporosis by demographic characteristics among patients with or without stroke.

Variables	Patients with stroke			Patients without stroke			Compared with non-stroke group		
	Osteoporosis	PY	Rate^†^	Osteoporosis	PY	Rate^†^	IRR (95% CI)^‡^	Adjusted HR (95% CI)^§^	*P*-value^¶^
All	1537	4662.48	32.97	5830	408189.5	14.28	2.31 (2.18 to 2.44)***	1.82 (1.71 to 1.94)***	
**Gender**									
Men	529	29008.03	18.19	1567	261343.15	5.99	3.03 (2.75 to 3.35)***	2.80 (2.49 to 3.16)***	<0.001
Women	1008	17534.45	57.49	4263	146846.35	29.03	1.98 (1.85 to 212)***	1.55 (1.44 to 1.68)***	
**Stratify age**									
50–59	303	14289.87	21.2	696	125926.86	5.53	3.84 (3.35 to 4.39)***	2.79 (2.42 to 3.22)***	<0.001
60–69	608	17536.24	34.67	2078	141367.58	14.69	2.36 (2.15 to 2.58)***	1.77 (1.61 to 1.95)***	
>70	626	14796.37	42.31	3056	140895.06	21.69	1.95 (1.79 to 2.13)***	1.61 (1.47 to 1.76)***	

***p < 0.001.

^†^Incidence rate in per 1000 personyears.

^‡^IRR in per 1000 person- -years.

^§^Model adjusted for age, sex, relevant comorbidities and medication.

^¶^*P*-value for interaction.

IRR, incidence rate ratio; PY, person-years.

Most patients have mild hemiplegia after stroke, and the reason for the increased risk of OF in stroke patients is related to osteoporosis, decreased bone mineral density and increased risk of falls on the hemiplegic side (4). In addition to increasing the risk of falling due to the loss of mobility of hemiplegic limbs, it can also lead to the reduction of stress stimulation received by bones and the increase in the functional activity of osteoclasts, which in turn leads to bone loss. Studies have confirmed that within 1 year after a stroke, the bone mineral density of the hemiplegic side will drop by 12–17% (5). In addition, stroke in some vascular areas of the brain stem (6) may lead to visual, motor, sensory or cognitive function, balance damage, and may also lead to falls, thus increasing the risk of fracture. Malnutrition, decreased sun exposure and subsequent vitamin D deficiency will all aggravate the bone loss in stroke patients; Common treatment methods for ischemic stroke, such as oral anticoagulants, may also increase the risk of fracture. In a word, as one of the common complications of stroke, fracture can further hinder functional recovery, prolong disability and increase the risk of death. Therefore, it is imperative to formulate prevention strategies for osteoporosis and fractures for stroke survivors (7).

Numerous scholars have conducted research on this disease before, covering the etiology, pathogenesis and treatment of post-stroke osteoporosis, and all of them have achieved good results. This paper systematically summarizes the etiology, pathogenesis and treatment methods of post-stroke osteoporosis by summarizing previous studies, and innovatively introduces the role of oxidative stress in the whole process of the disease, which provides new ideas for the related research and treatment of the disease in the future.

## 2. The etiology and mechanism of osteoporosis after stroke

**FIGURE 1:**
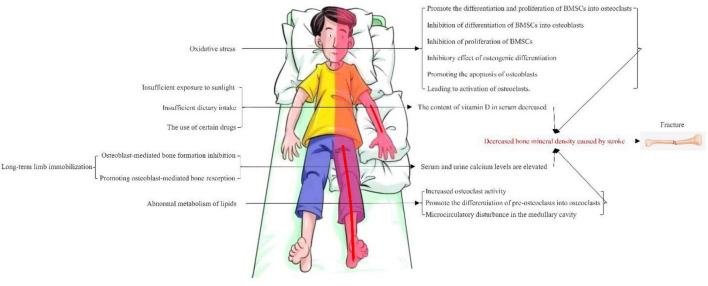
Multiple pathogenic mechanisms of osteoporosis after stroke.

Osteoporosis is a known consequence of stroke. The pattern of bone loss observed in patients with stroke is different from that usually encountered with postmenopausal osteoporosis, which is limited to the paralyzed side and more obvious in the upper limbs. There are many reasons for osteoporosis in stroke patients, including limited exercise and reduced load due to paralysis, insufficient nutrition intake due to eating disorders, intake of various drugs and reduction of vitamin D due to insufficient sunshine (8), As shown in Figure 1. The pathogenesis of post-stroke osteoporosis is not clear. Mild paralysis, decreased mobility and decreased bone load seem to play a major role, and other factors such as nutrition and iatrogenic factors may also play an important role (9).

### 2.1. Osteoporosis after stroke and bone density

People at high risk of stroke are already at risk for osteoporosis and fracture (10). However, research on changes in BMD in patients with osteopenia after stroke is limited, and the research on the appropriate treatment and management of osteopenia is also rare. In addition, there are few studies to compare the changes in BMD in stroke patients with osteoporosis and stroke patients with osteoporosis. According to the World Health Organization, the BMD T of −2.5 or less is a defining characteristic of osteoporosis. Several studies have investigated the relationship between low BMD and stroke, suggesting that low BMD is a potential risk factor for stroke and affects the long-term prognosis of stroke (10–13). Osteoporosis is a metabolic bone disease characterized by an imbalance between bone resorption and bone accumulation, resulting in micro-architectural disruption, decreased BMD and increased bone fragility (14). Accelerated loss of in bone mineral density after stroke (15, 16) can lead to fractures in stroke survivors, and post-stroke weight-bearing limitation of the affected limb inevitably leads to bone loss. After a stroke, most fractures occur on the hemiplegic side of the body because the BMD of that side is 4.6–14% lower than that of the uninjured side (8). In addition, social deprivation, malnutrition, reduced sun exposure and subsequent vitamin D deficiency accelerate bone loss in stroke survivors (4). Bone loss starts immediately after the stroke, lasts for 3–4 months after the stroke, and continues at a slower rate for up to a year after the stroke (17). In addition, stroke patients may have various neurological deficits that can lead to reduced physical activity and reduced use of a paralyzed limb, which can lead to bone loss. Although researchers have not yet definitively established whether osteoporosis is a hallmark of stroke, a potentially complex causal relationship between stroke and osteoporosis has been reported (18).

### 2.2. Osteoporosis after stroke and limb immobilization

There are many potential mechanisms that lead to bone loss after stroke, and restricted movement is one of the important factors involved in this process. BMD studies of the upper and lower extremities consistently show more bone loss on the restricted side than on the unrestricted side. The exact mechanism of the reduction in BMD on the hemiplegic side after stroke is not fully understood, but the link between reduced mobility and bone loss has long been known. In a pivotal study published by Schneider and McDonald in 1984, serum and urinary calcium rose rapidly in 90 healthy young men on bed rest for 5–36 weeks, plateaued for several weeks at week 6, and then declined to a plateau above the dynamic baseline. This happened even when volunteers were given vitamin D supplements throughout the study (19). Reduction of mechanical stress on bone inhibits osteoblast-mediated bone formation and accelerates osteoblast-mediated bone resorption, leading to disuse osteoporosis, mechanical stress on bone is a determinant of bone morphology, BMD and bone strength One of the factors is therefore that disuse accelerates bone resorption and inhibits bone formation, making bones atrophic and brittle (20). This explains why disability after stroke is related to abnormal motor function. It has been shown that bone mineral loss is more pronounced in the upper extremities than in the lower extremities, and in long-term hemiparesis after stroke, the difference between the two sides is more pronounced. It is worth noting that the BMD of the non-hemiplegic side is between that of the hemiplegic side and the normal side. Due to the stroke, his activities of daily living require assistance and the mobility of the whole limb is reduced, resulting in a wide range of mild osteoporosis, severe osteoporosis The degree is consistent with the degree of limitation of the patient’s overall activity (21). However, in some stroke patients, the use of the paralyzed arm in the upper limb is greatly reduced, consistent with a large decrease in bone mass, which is compensated for by increased use of the non-paralyzed arm, which may result in no loss of bone mass and may even increase (22). The degree of bone loss depends on age, severity of hemiplegia, duration of total or partial immobilization, and the degree of reduction in bone loading on the affected side and reduction in muscle stretch. Not only may these factors act differently in the upper and lower extremities, but they may also act differently in trabecular and cortical bone for the same bone, as shown by Liu et al. (23). Some studies have also shown that men who have had a stroke have a higher prevalence of osteopenia and osteoporosis and less moderate or vigorous physical activity than men without a stroke. In women, BMD is not associated with stroke (24).

### 2.3. Post-stroke osteoporosis and serum vitamin D

Vitamin D is one of the fat-soluble vitamins thought to have anti-rickets activity. Vitamin D is not only abundant in nature, but is also synthesized in the skin with the help of the sun’s UV-B rays (25). Vitamin D deficiency and concomitant arterial disease may contribute to increased severity and depth of bone loss. Vitamin D deficiency is also common after a stroke and can be caused by insufficient sunlight, but also by dietary deficiencies (26). After stroke, an inflammatory response may also be triggered, affecting intestinal absorption of vitamin D and metabolism of vitamin D in the liver, thereby reducing serum vitamin D levels. In a study of 152 people by Wang, Q et al., serum vitamin D levels in acute stroke patients were correlated with inflammatory markers, including hsCRP, white blood cell count, neutrophil-γ/-γ lymphocyte ratio, IL-γ6 and TNF-α: They found that vitamin D levels were negatively correlated with serum IL-β6 levels and hsCRP, whereas serum vitamin D levels were not correlated with other inflammatory markers such as white blood cell count, neutrophil-γ/-γ lymphocytes and TNF-α. In another observational study of 957 older adults, there was no association between vitamin D and TNF-α levels (27). Some medicines can affect the metabolism of vitamin D, including anti-epileptic medicines, glucocorticoids, etc. These drugs can cause vitamin D deficiency and reduce serum vitamin D levels. Therefore, physical status and treatment regimen after stroke may affect serum vitamin D levels.

### 2.4. The relationship between lipid metabolism and bone metabolism after stroke

Osteoporosis is closely linked to hyperlipidemia and its incidence increases with age. This may be due to an imbalance between bone cells and fat cells in the bone marrow. Research shows that among stroke patients admitted to rehabilitation, the proportion of patients with abnormal bone density is higher than that of those with normal bone density. The levels of total cholesterol, high-density lipoprotein (HDL), low-density lipoprotein (LDL), apolipoprotein A and B in the abnormal group were higher than those in the normal group, while the bone mineral density of L1 ∼ L4, femoral neck and proximal femur were lower than those in the normal group, suggesting that the level of lipid metabolism in stroke patients may be the influencing factor of their bone (28), Other studies have found that total cholesterol and triacylglycerol are risk factors for decreased bone mineral density in stroke patients, and that disruption of lipid metabolism increases osteoclast activity. A high-fat diet may promote the differentiation of precursor osteoclasts into osteoclasts, increasing the volume and activity of osteoclasts, thereby promoting bone resorption and increasing the risk of osteoporosis (29). Second, the disruption of lipid metabolism leads to microcirculatory disturbances in the bone marrow cavity. In the hyperlipidemic rat model, the proportion of adipose tissue in the bone marrow cavity increased significantly, while the proportion of blood sinus tissue decreased. Fat accumulation also increases the pressure of the bone space and compresses the blood vessels in the cavity, eventually leading to inadequate blood supply to bone tissue, reduced bone marrow microcirculation, reduced osteogenesis and ultimately reduced bone density (30).

### 2.5. Osteoporosis after stroke caused by medication

Proton pump inhibitors (PPIs) are powerful acid-suppressing drugs that are currently widely used to treat drug-related upper gastrointestinal disorders. Current research suggests a link between the use of PPIs and the risk of osteoporosis and bone fractures (31–33). However, not all studies support this link (34, 35). In stroke survivors, antiplatelet drugs are commonly used for secondary prevention of stroke. However, antiplatelets have adverse effects on the upper gastrointestinal tract, ranging from heartburn and gastroesophageal reflux disease (GERD) to severe stomach ulcers (36). These adverse effects may lead to poor compliance or even discontinuation of antiplatelet therapy, which may ultimately lead to recurrence of ischemic stroke. Therefore, it is recommended that stroke patients who require continuous antiplatelet therapy use a PPI for gastric protection at the same time. In addition, hypertension is an important modifiable risk factor for preventing stroke recurrence, but previous studies have shown that patients treated for hypertension have an increased risk of GERD (37). In addition, reduced lower esophageal sphincter pressure is common in stroke patients, which may further contribute to GERD. Therefore, in clinical practice, a significant proportion of stroke patients receive PPI therapy (38). In the study by Lin et al. (39), The incidence of osteoporosis, hip fracture and cone bone fracture were higher in patients using PPIs than in those not using PPIs, and the incidence of osteoporosis, hip fracture and cone bone fracture increased with increasing PPI dose. The exact biological mechanism for the association between PPIs and the risk of osteoporosis and fractures is unclear. One possibility is that PPIs may reduce calcium absorption. Since the acidic environment of the stomach promotes the dissociation of insoluble calcium salts into calcium ions, PPIs act as potent inhibitors of gastric acid secretion, which may affect calcium absorption. In addition, PPIs may also affect the function of osteoblasts and osteoclasts, interfering with osteoblast function by inhibiting tissue non-specific phosphatase in osteoclasts, and with osteoblast and osteoclast function by inducing MEK and JNK pathways. In addition, PPIs have been linked to malabsorption of vitamin B12, which can lead to hyperhomocysteinemia, which affects collagen cross-linking, resulting in reduced bone strength (39).

Post-stroke depression is very common, with a reported prevalence of approximately 31% (40). Which is higher than the prevalence of depression before stroke (11.6%). Depression can be effectively treated with antidepressants, most commonly selective serotonin reuptake inhibitors (SSRIs) (41). In the study by Jones, JS et al. (42) found that the risk of fractures doubled within 6 months after a stroke, but there was no significant effect on the risk of falls, seizures or stroke recurrence. However, the above trials only confirmed that fluoxetine and citalopram may increase the risk of fracture in stroke patients and cannot be extended to other SSRIs. In addition, the mechanism by which SSRIs increase the risk of fracture is unclear, and it is uncertain whether the duration of treatment is a predictor of fracture. However, in the study by Richter et al., the proportion of falls due to injury did not increase with SSRI treatment, and the decrease in bone mineral density with SSRI treatment may explain this finding (43). It should be noted that the above only discusses the relationship between the use of antidepressants and osteoporosis after stroke from one point of view. As this relationship may be influenced by many factors, it is necessary to investigate the relevant mechanism from many aspects, such as the sex, age, type of drug and dosage of the patient.

## 3. Oxidative stress and stroke

Cardiovascular and cerebrovascular diseases are among the leading causes of death, accounting for about 40% of deaths in China (44). Atherosclerosis is a chronic inflammatory disease characterized by the accumulation of lipids and inflammatory cells in the walls of large and medium-sized arteries. The pathogenesis of atherosclerosis involves the activation of pro-inflammatory signaling pathways, the expression of cytokines/chemokines and an increase in oxidative stress, an imbalance that is conducive to an increase in reactive oxygen species (ROS) production and/or a decrease in the innate antioxidant defense system *in vivo* (45). This process is long and involves many pathological processes, and the causes and molecular biological mechanisms are not fully understood. In recent years, however, much evidence has emerged that oxidative stress plays an important role in the development and progression of atherosclerosis.

### 3.1. Homocysteine (Hcy) and stroke

Studies have shown that serum homocysteine (Hcy) is an independent risk factor for acute cerebral infarction, and that changes in serum Hcy levels are closely related to changes in cerebral vascular endothelial function and the occurrence of cerebral infarction (46). Hcy is a normal metabolite in the human body. When its level rises, it damages the vascular endothelium, affects the proliferation of smooth muscle cells and reduces the levels of NO and endothelial nitric oxide (eNOS) (47), disrupt the endothelial function of cells (48). This leads to atherosclerosis and/or plaque formation in the head and neck in turn, and ultimately to stroke. Nitric oxide synthase (NOS) plays an antioxidant and pro-oxidant role in atherosclerosis. eNOS is structurally expressed in endothelial cells. NO produced by activation of eNOS can inhibit low-density lipoprotein (LDL) oxidation, white blood cell adhesion and migration, vascular smooth muscle cell (VSMC) proliferation and platelet aggregation. (49).

In addition to the oxidative stress mechanism, Hcy may also play a neurotoxic role through microglia-mediated neuroinflammatory injury: treatment with Hcy can activate microglia, significantly increase the volume of cerebral infarction and induce cell injury. One possible mechanism by which Hcy enhances the inflammatory response of microglia is that JAK 2/STAT 3, a key immune signaling pathway, is normally expressed in the brain. It also plays an important role in regulating microglial activation and inflammatory response. The study by Chen S et al. tested whether the JAK 2 inhibitor AG 490 could affect the expression of pSTAT3, the microglia-specific markers Iba-1 and OX-42, and the pro-inflammatory mediators TNF-α and IL-6 induced by Hcy. The results showed that activation of STAT 3 and secretion of IL-6 in microglia were significantly upregulated in Hcy-treated ischemic brain tissue and this effect was reversed by AG 490. The experimental results indicate that the increased expression of STAT 3 after homocysteine treatment may be involved in microglial activation and neuroinflammatory injury in rats with middle cerebral artery ischemia-reperfusion.

For bone, there is limited evidence that homocysteine has a direct effect on bone, including bone density. The potential mechanism of the association between high homocysteine levels and fracture risk may involve the interference of homocysteine with collagen cross-linking and the specific interference of homocysteine with collagen cross-linking and fibril formation in solution. As collagen cross-linking is very important for the stability and strength of the collagen network, interference with its formation may alter the bone matrix, which may increase bone fragility (50).

### 3.2. Ferroptosis and ischemia-reperfusion injury after stroke

When blood flow is restored after cerebral ischemia, brain tissue can be damaged and deteriorate. Many mechanisms are involved in this process, such as inflammatory activation, oxidative stress, ferroptosis, etc. Among these, dysregulation of iron metabolism is closely linked to the occurrence of ischemia-reperfusion injury (50): after reperfusion, excessive reactive oxygen species (ROS) and iron accumulation mediate ischemia-reperfusion injury after stroke. (1) The Fenton reaction occurs between the cytoplasm, mitochondria and excess free iron to generate hydroxyl radicals, which have toxic effects. With calcium ion overload, the mitochondrial respiratory chain is blocked and neurons die. (2) Lipids induce excessive ROS production through the Fenton reaction. ROS mainly include oxygen molecules, hydroxyl radicals, superoxide anions and hydrogen peroxide radicals, and excessive ROS will destroy cell homeostasis. ROS are involved in oxidative stress, promoting lipid peroxidation, depleting the antioxidant capacity of cells and causing superoxide damage to the inner wall of cerebral arteries. At the same time, Fe^2+^ and Fe^3+^ mediate lipid peroxidation through hydroperoxide to form lipid free radicals, leading to DNA denaturation and further aggravating cell damage. (3) The accumulation of ROS can cause a large increase in macrophages, damage to the blood-brain barrier, hemorrhagic transformation and cerebral edema after stroke.

### 3.3. Oxidative stress/nitration stress and cerebral atherosclerosis

Oxidative and nitrosative stress are characterized by an imbalance between oxidative and antioxidant systems, leading to an increase in reactive oxygen species (ROS) and reactive nitrogen species (RNS). The vascular wall is also equipped with oxidative systems such as xanthine oxidase. (51), mitochondrial respiratory chain enzyme (52), Lipoxygenase (53), uncoupled eNOS (54), Nicotinamide adenine dinucleotide phosphate hydrogen oxidase (NADPH oxidase) (55), and antioxidant systems, including Superoxide dismutase (SOD), Catalase Glutathione peroxidase, Paraoxonase (PON), thioredoxin system, and peroxiredoxin (56).

Among these, NOx (NADPH oxidase) is considered to be an important source of RONS in the vascular wall, and there are many types of NOx, of which NOx 4 is the most abundant at the vascular level, but it is controversial whether it has the functions of promoting atherosclerosis and preventing atherosclerosis at the same time: NOx 4 releases more hydrogen peroxide than O^2–^ (57), so amount of peroxynitrite (ONOO-) formed is therefore low and the bioavailability of NO is maintained; other studies have shown that the increase in NOx 4 activity destroys vascular function in some diseases, such as diabetic cardiomyopathy (58). The Increased NOx activity leads to eNOS uncoupling, decreased NO bioavailability and endothelial dysfunction. Uncoupled eNOS exhibits Nox activity and produces O^2–^, exacerbating oxidative stress in the vascular wall. Neuronal NO synthase (nNOS) is expressed in central and peripheral nerve cells and blood vessel walls, which contributes to vasodilation and is considered anti-atherosclerotic. In contrast, inducible NOS (iNOS), which is induced by inflammation, oxidative stress and sepsis, is atherosclerotic, possibly due to the formation of peroxynitrite (ONOO^–^), thereby increasing nitrite stress (59).

## 4. Oxidative stress and osteoporosis after stroke

After a stroke, dyskinesia reduces the activity of the limbs and bone tissue loses the stimulus of mechanical stress. When the mechanical stress received by the bone is reduced, the activity of osteoclasts is increased, which leads to the easy absorption of bone tissue and the obvious reduction of bone mineral density in the body, which eventually leads to different degrees of osteoporosis. Increasing daily activity can reduce the incidence of osteoporosis, while immobilization after bed rest can cause hypercalcemia and hypercalciuria in patients with moderate and severe stroke, especially in the elderly, and accelerate bone absorption to cause osteoporosis.

### 4.1. Oxidative stress and osteoporosis

Mitochondria, the powerhouses of cells, maximize the use of intracellular oxygen while generating energy and ROS. Low levels of ROS can maintain bone homeostasis and balance between osteoclasts and osteoblasts (60). Abnormal ROS levels have been shown to lead to the death of osteoblasts and osteoclasts and the reduction of bone structure (61). Oxidative stress has been shown to shorten the lifespan of osteoblasts in mouse models of osteoporosis and to reduce trabecular bone density. Oxidative stress can promote the differentiation and proliferation of bone marrow mesenchymal stem cells (BMSCs) into osteoclasts. Bone marrow mesenchymal stem cells are bone marrow-derived progenitor cells that can differentiate into osteoblasts, chondrocytes, adipocytes, myoblasts and other cells. The dynamic balance of osteogenic differentiation, apoptosis and metabolism of bone marrow mesenchymal stem cells plays a key role in maintaining bone tissue structure and bone mass homeostasis. The lack of differentiation capacity of bone marrow mesenchymal stem cells is one of the mechanisms leading to osteoporosis. Oxidative stress can inhibit osteogenic differentiation and damage bone marrow mesenchymal stem cells. During oxidative stress, excessive accumulation of active oxygen in the body will have a negative effect on the differentiation of bone marrow mesenchymal stem cells into osteoblasts. This peroxidation not only damages molecular structures such as proteins, lipids, deoxyribonucleic acid (DNA) and cell structures such as mitochondria and endoplasmic reticulum, but also inhibits the ability of the bone marrow mesenchymal stem cells themselves to proliferate (62). The increase in ROS and the decrease in antioxidant levels lead to an increase in osteoclast activity and a decrease in the osteogenic potential of osteoblasts, resulting in bone degradation.

### 4.2. The negative effects of oxidative stress on osteoblasts

The maintenance of bone mass depends not only on the resorptive function of osteoclasts and the function of osteoblasts, but also on the difference in the production and apoptosis rates of osteoblasts and osteoclasts. Among them, osteoblasts play an important role in maintaining bone homeostasis, regulating cytoplasmic matrix mineralization, controlling bone remodeling and osteoclast differentiation (63). Osteoblast apoptosis promotes the development of osteoporosis, so inhibiting osteoblast apoptosis provides a new direction for the prevention and treatment of osteoporosis. Studies have shown that oxidative stress plays an important role in the pathological process of osteoporosis (64). During the pathogenesis of osteoporosis, osteoblast oxidative stress levels increase significantly, suggesting that osteoblast oxidative stress plays a critical role in pathological bone loss. Oxidative stress not only inhibits osteogenic differentiation but also promotes osteoblast apoptosis (65). Therefore, it is of great importance to explore the mechanism of oxidative stress-induced osteoblast apoptosis to understand the pathogenesis of osteoporosis. Osteoclast is a type of multinucleated cell derived from the monocyte/macrophage lineage and is the only cell with bone resorption capacity. Oxidative stress can activate the differentiation of osteoclast precursors and increase bone resorption, as shown by the increase in tartrate-resistant acid phosphatase (TRAP) activity in osteoclasts and the increase in bone resorption area on the bone surface (66). Autophagy is a catabolic process, which removes damaged organelles and some cell molecules including protein aggregates through lysosomal digestion (67). More and more evidence show that autophagy dysfunction leads to changes in osteoclast function and increased bone loss. Under oxidative stress, autophagy is activated, accompanied by an abnormal increase in osteoclast differentiation and bone resorption. Therefore, the interaction between oxidative stress and autophagy plays an important role in intracellular homeostasis and osteoclast survival. Therefore, inhibition of autophagy may delay osteoclast activation caused by excessive ROS. (68).

## 5. Treatment of osteoporosis after stroke

The management of osteoporosis post-stroke warrants meticulous attention. Osteoporosis significantly heightens fracture risk, and post-stroke patients typically require prolonged bed rest or wheelchair use, which may expedite bone loss. Hence, timely and effective treatment measures for stroke patients with osteoporosis become crucial. At present, the management of osteoporosis post-stroke encompasses drug therapy, calcium and vitamin D supplementation, exercise, and rehabilitation. The medication regimen typically involves estrogen drugs, bisphosphonates, and biodegradable, osteocalcin-related peptides which have shown efficacy in reducing bone loss rate and fracture incidence. Calcium and vitamin D supplementation can improve bone mineral density, while exercise and rehabilitation can lower the risk of fracture by enhancing muscle strength and balance. Additionally, the writing must adhere to grammatical correctness, avoid biased language, and maintain clear, concise, and objective language throughout the text. However, it must be noted that subjective evaluations need to be excluded unless clearly marked as such. The treatment plan needs to be tailored to the individual conditions of each patient, and therefore, an individualized treatment plan should be created accordingly. Abbreviations of technical terms must always be explained when they are first used. In brief, the comprehensive consideration of several factors is crucial in addressing osteoporosis treatment after stroke. An individualized treatment plan is useful in mitigating fracture risk and enhancing patients’ quality of life. However, a study of stroke patients revealed inadequate assessment and treatment of osteoporosis and fracture risk factors. However, a study of stroke patients revealed inadequate assessment and treatment of osteoporosis and fracture risk factors. However, a study of stroke patients revealed inadequate assessment and treatment of osteoporosis and fracture risk factors. The percentage of stroke patients receiving osteoporosis medication or supplements is relatively low. It is essential to enhance comprehension, prevention, and management of bone loss in this vulnerable population (69).

Non-pharmacological treatment remains the cornerstone for maintaining bone health prior to the onset of osteoporosis or fragility fractures. Physical exercise is particularly advantageous in averting falls and fractures among the wider population (70–72). Gait defects and long-term immobilization are the primary risk factors for osteoporosis development after a stroke. Therefore, it is crucial to implement strategies to improve these issues. Utilizing techniques to enhance walking ability and interaction between lower limbs and the ground will limit bone loss. It is imperative to avoid biased and ornamental language while complying with standard grammatical rules and technical vocabulary. Treatment must aim to enhance motor function and strength, rectify walking-limiting deformities, and suppress spasms. Consistency in formatting and citation style is vital as well. Aerobic recovery and weight-bearing exercise may enhance bone mass in individuals suffering from chronic stroke.

Another non-pharmacological approach involves increasing the duration of exposure to sunlight. Stroke patients frequently face social isolation, which may restrict their exposure to sunlight. That is one of the reasons behind the vitamin D deficiency in stroke patients. The scientific comprehension of the significance of vitamin D extends beyond its role in the absorption of calcium and phosphorus and the maintenance of healthy teeth and bones. Especially amongst the elderly, vitamin D assumes a crucial physiological function in numerous non-skeletal processes, such as regulating the normal thyroid function, blood clotting, providing muscle strength and flexibility, enhancing the production of endogenous antibiotics, preventing the onset of autoimmune and allergic diseases, combating infectious diseases, and discouraging the growth of tumors (73–81). According to the National Institutes of Health (NIH) recommendations, adults under the age of 70 should consume 15 μg (600 IU) of vitamin D daily, whereas those above 70 should consume 20 μg (800 IU). The body can produce vitamin D3 (cholecalciferol) by exposing the skin to UVB light and obtain provitamin D2 (ergocalciferol) through consumption. D3 is the more dominant form. Exposing uncovered skin to sunlight for 20–30 min daily can fulfill your vitamin D requirements. Nevertheless, the skin’s capacity to generate vitamin D3 declines with age (82). This is one reason why stroke survivors may have insufficient levels of vitamin D. Making certain that these individuals receive adequate sun exposure daily is crucial in the prevention and treatment of osteoporosis after a stroke. In a study conducted by Hsieh, CY et al., it was suggested that postmenopausal women take supplements of both calcium and vitamin D to decrease the risk of fractures in those individuals who have osteoporosis and stroke (83). As vitamin D deficiency is a frequent occurrence following a stroke, administering vitamin D supplements (at a dosage of 800 to 1000 U/day) may prove advantageous in preventing fractures among patients with stroke and osteoporosis (84). In addition, vitamin D deficiency in stroke patients may cause various issues. For instance, it can lead to impaired absorption of calcium in the gastrointestinal tract, compromised bone mineralization and muscle strength, and is associated with decreased muscle mass, consequently increasing the risk of falls (85). Vitamin D also possesses neuroprotective, neuromuscular, and skeletal protective effects, potentially mitigating the cognitive and functional damage experienced by patients who have suffered a stroke (86). A lack of Vitamin D can cause mild secondary hyperparathyroidism. Mild deficiency of Vitamin D can result in “type II” osteoporosis, which can cause hip fractures in individuals aged approximately 70, both male and female. Inpatients and outpatients after a stroke commonly have reduced vitamin D intake through their diet and reduced exposure to sunlight. In cases where elderly hospitalized stroke patients are at risk of deficiency, most should be given vitamin D3 (800–2000 U/day) and calcium supplements. This is particularly important for long-term stroke patients. Vitamin D3 and calcium supplementation can reduce hip fracture by up to 43% in elderly hospitalized women (average age 84 years old), who are not selected for stroke. Nevertheless, compared with placebo, there is no significant decrease in hip fracture incidence for elderly hospitalized stroke patients with vitamin D3 and calcium supplements. However, the absence of active vitamin D [1 1, 25 (OH) 2D] may also contribute to bone loss resulting from stroke. Nevertheless, there is insufficient evidence to make any definitive statements. More research is required to substantiate the consequences of calcium and vitamin D supplements on the skeletal and extra-skeletal components of stroke sufferers (87). A study conducted by Uluduz, D et al. suggests that vitamin D insufficiency could be another reason for the considerable prevalence of osteoporosis in stroke survivors. Inadequate quantities of vitamin D can decrease bone mineral density, cause neuromuscular impairment and heighten the likelihood of falls and fractures (88, 89). In a long-term study of stroke survivors, a correlation has been found between low levels of vitamin D, low bone mineral density, and hip fractures post-stroke (90). They discovered that stroke survivors had a greater incidence of osteoporosis compared to non-stroke participants and a higher incidence of vitamin D deficiency. However, there was no direct link established between vitamin D deficiency and osteoporosis. Bone loss occurs in the initial stages post-stroke and escalates over time due to restricted physical activity. Existing data imply that other factors, like inactivity or osteoporosis itself, instead of vitamin D deficiency, may serve as explanations. The elevated prevalence of vitamin D deficiency in stroke survivors may be associated with malnutrition or decreased exposure to sunlight and skin production. Further research is required to assess the clinical importance of vitamin D deficiency in stroke survivors (91). A meta-analysis has indicated that daily intake of vitamin D and calcium cannot be strongly recommended to prevent fractures due to methodological concerns. Furthermore, the efficacy and safety of high-dose vitamin D in high-risk groups is uncertain (92). The previous research solely discusses the role of physical therapy in averting bone loss following a stroke. Further areas, including nutrition and vitamin supplements, have not been examined in enhancing bone health (93). Only one study, conducted by Han et al. (94) ensured that all participants received sufficient protein, vitamin D and calcium in their diet. However, the study did not mention monitoring intake through methods such as pill counting or food diaries. None of the studies included in the review monitored serum 25 (OH)D levels to detect vitamin D deficiency. In a Rotterdam-based study, vitamin D deficiency was discovered to be a result of stroke, which quickened the loss of proximal femur bone in post-stroke patients. The risk factors for osteoporosis following a stroke include physical inactivity, malnutrition, illness, and ageing, which all increase the likelihood of falls and fractures. The deterioration of muscle strength and mass after an acute stroke necessitates early intervention to maintain the bone density/strength index. Therefore, this must be addressed promptly as a functional unit. Acute stroke induces muscle hyper catabolism, which results in greater protein degradation than synthesis. Studies have demonstrated the ability of amino acid supplementation to reverse the effects of stroke. However, the impact that amino acid supplementation has on bone properties was not measured. Therefore, in order to create the best prevention and treatment strategy for bone loss and osteoporosis after stroke, it is advisable to adopt an array of interventions, including individualized physical and drug therapy, ample intake of protein, calcium and vitamin D, rather than relying on a single approach or treatment (95).

Regarding the effect of statins on osteoporosis after stroke, Lin, SM and colleagues (7) conducted a population-based trend-matching cohort study and concluded that statin use was associated with a decreased risk of osteoporosis, hip fracture, and vertebral fracture in stroke patients. Furthermore, a dose-response relationship between statin cDDD and reduced risk of osteoporosis and fractures was observed. A recent meta-analysis, encompassing clinical trials and observational studies, demonstrated a significant correlation between statin consumption and augmented bone mineral density alongside mitigated hip fracture hazard. Users of statin exhibited a lower odds ratio of 0.75 than non-users, with a consequent risk reduction of vertebral fracture (OR = 0.81), albeit the tendency was deemed statistically insignificant (96). Another meta-analysis also found a noteworthy correlation between the use of statins and the reduction of overall fracture risk, with an odds ratio of 0.80 (97). On the other hand, a recent meta-analysis specifically examining clinical trials found that although statin use was associated with an increase in BMD, there was no statistically significant correlation with fracture risk (98). The link between statins and osteoporosis and fracture risk remains unclear. However, some pathways through which statins may affect bone metabolism have been identified in prior research. Specifically, statins can upregulate bone morphogenetic protein-2 via the ras/phosphoinositide 3-kinase/protein kinase B/mitogen-activated protein kinase signal pathway. This, in turn, stimulates the expression of runt-related transcription factor 2, inducing osteoblast differentiation. The proliferation and differentiation of osteoblasts can be promoted by statins through inhibiting the synthesis of farnesyl pyrophosphate and geranyl pyrophosphate. Technical term abbreviations such as SMAD will be explained when first used. Furthermore, statins regulate the transforming growth factor-b/SMAD 3 signaling pathway to prevent apoptosis in osteoblasts. In addition, statins could potentially activate the expression of estrogen receptor-α via the osteoprotegerin/nuclear factor kB receptor activator ligand/nuclear factor kB receptor activator signaling pathway while simultaneously impeding the generation of osteoclasts, resulting in augmented bone formation (7). In summary, the utilization of statins is correlated with a decrease in the risk of osteoporosis, hip fracture, and vertebral fracture among stroke patients, with a noted dose-effect relationship. However, additional prospective clinical trials are necessary to reaffirm these findings, which can aid in the development and application of pharmaceuticals.

Bisphosphonates are frequently used as therapeutic drugs for osteoporosis following stroke, and previous studies have reported their beneficial effects on stroke patients (99), Bisphosphonate therapy proves effective in diminishing the occurrence of spinal fracture subsequent to stroke by enhancing lumbar spine bone mineral density (LS BMD). A study indicates that bisphosphonates reduce the risk of spinal fractures by 35–50% and improve LS BMD by 1–6%. Given the literature indicating a correlation between decreased physical activity due to osteoporotic spinal fractures and increased risk of stroke, treating osteoporosis can serve as a preventative measure for stroke recurrence. Furthermore, this study indicates that bisphosphonate use in the osteoporosis group significantly prevents a decrease in femoral neck (FN BMD) compared to the osteopenia group. One such bisphosphonate is zoledronic acid salt. In Poole’s study and others, an iliac bone biopsy was obtained within 3 months after an acute stroke and compared to a healthy control group. The study found a decrease in bone formation at the tissue level regardless of whether the patient was treated with Zoledronic acid salt or placebo. The number of osteoclasts in patients treated with zoledronate was lower than in the control group treated with a placebo (100). In the Poole, KE et al. study, the use of zoledronic acid was found to prevent bone mineral density (BMD) loss in the hemiplegic hip joint. Administered intravenously within 5 weeks of admission, the treatment effectively prevented bone loss in patients with hemiplegia who were unable to walk independently for at least a week after a stroke. This study was the first to confirm the effectiveness of intravenous bisphosphonates in preventing significant bone loss in the hemiplegic hip joint during the first year. They discovered that stroke patients treated with zoledronic acid had stable average femoral neck BMD within 12 months, with a minute 0.1% change (95% CI, 2.5, 2.7). Thus, zoledronate is an effective countermeasure to prevent hemiplegic hip joint bone loss in acute stroke treatment. Other research demonstrated a significant increase in osteoclast absorption within 1 week of acute stroke according to biomarkers (101), Zoledronic acid salt remains a strong and enduring suppressor of osteoclast resorption even after a year. Supporting this, the histomorphometry analysis of biopsy samples taken from the iliac bone of hemiplegic patients in the Zoledronic acid salt group confirmed a marked decrease in the number of osteoclasts and their progenitor cells. It should be noted that the utilization of zoledronate can result in adverse gastrointestinal reactions and renal function impairment. Therefore, caution should be exercised while administering this medication.

## 6. Discussion

Fracture is now recognized as a risk factor in stroke, and preventing the development of hemiplegic osteoporosis should be a priority in managing stroke patients. Post-stroke osteoporosis has not been fully recognized and treated compared to postmenopausal osteoporosis. Oxidative stress theory is a crucial theory in the process of stroke and osteoporosis. It is important to investigate the mechanism of oxidative stress in the cardiovascular, cerebrovascular, and bone metabolism systems to elucidate the causes of stroke and osteoporosis. As its research deepens, our comprehension of the physiological and pathological mechanisms and treatment measures increases. Controlling the serum homocysteine level, avoiding or relieving ischemia-reperfusion of cerebrovascular or limb blood vessels, and managing atherosclerosis caused by nitration stress (rather than oxidative stress) through medication are all important for the treatment of stroke and the subsequent osteoporosis. In the future, treatment can focus on two areas: (1) Blocking signal pathways associated with oxidative stress during cerebral atherosclerosis or mitigating ischemia-reperfusion injury; and (2) Addressing the impact of lipid metabolism disorder or oxidative stress on osteoclast activity post-stroke. It is expected that advancements in molecular biology, basic medicine, and clinical medicine technology will enhance the comprehension of the pathogenesis of stroke-induced osteoporosis, and facilitate the development of more effective drugs and technologies to treat and prevent it. Osteoporosis after stroke is a prevalent and incapacitating aftermath that impairs the quality of life of stroke victims. The precise mechanism underlying the occurrence of stroke and osteoporosis is yet to be fully understood, hampering progress in developing effective intervention strategies. Nonetheless, recent research suggests that the incidence of fracture can be effectively reduced by mitigating the risk of falls through activities such as drug therapy, physical exercise and targeted fall-prevention measures. Further research is required to establish the most efficient intervention methods for osteoporosis in stroke survivors and to determine the means of resolving complications simultaneously.

This study provides an overview of the pathogenesis and treatment options for stroke and post-stroke osteoporosis. It also explores the role of oxidative stress theory in the disease, presenting information on its etiology, symptoms, treatment methods, and suggestions for preventive measures. The study examines treatment outcomes and side effects, and offers guidance to doctors in formulating more appropriate treatment plans. Furthermore, it presents novel ideas for developing new drugs or treatment methods in the future, with potentially significant impacts on the prevention and treatment of post-stroke osteoporosis patients.

## Author contributions

JL: Visualization, Writing – original draft, Writing – review and editing. JS: Conceptualization, Formal Analysis, Funding acquisition, Investigation, Methodology, Project administration, Resources, Supervision, Validation, Writing – review and editing. LS: Data curation, Investigation, Software, Writing – review and editing.

## References

[1] Lee KB Lee JG Kim BJ Lee K Han M Park J. The epidemiology of fracture in patients with acute ischemic Stroke in Korea. *J Korean Med Sci.* (2019) 34:e164. 10.3346/jkms.2019.34.e164 31172697 PMC6556443

[2] Pouwels S Lalmohamed A Leufkens B de Boer A Cooper C van Staa T Risk of hip/femur fracture after stroke: a population-based case-control study. *Stroke.* (2009) 40:3281–5. 10.1161/STROKEAHA.109.554055 19661475

[3] Lisabeth LD Morgenstern LB Wing JJ Sanchez BN Zahuranec DB Skolarus LE Poststroke Fractures in A Bi-ethnic Community. *J Stroke Cerebrovas Dis.* (2012) 4. 10.1016/j.jstrokecerebrovasdis.2010.11.009 21334222 PMC3167994

[4] Wang HP Sung SF Yang HY Huang WT Hsieh CY. Associations between stroke type, stroke severity, and pre-stroke osteoporosis with the risk of post-stroke fracture: a nationwide population-based study. *J Neurol Sci.* (2021) 427:117512. 10.1016/j.jns.2021.117512 34082148

[5] Benzinger P Rapp K Konig HH Bleibler F Globas C Beyersmann J Risk of osteoporotic fractures following stroke in older persons. *Osteoporos Int.* (2015) 26:1341–9. 10.1007/s00198-014-3005-x 25572044

[6] Razek A Elsebaie NA. Imaging of vascular cognitive impairment. *Clin Imaging.* (2021) 74:45–54. 10.1016/j.clinimag.2020.12.038 33434866

[7] Lin SM Wang JH Liang CC Huang HK. Statin use is associated with decreased osteoporosis and fracture risks in stroke patients. *J Clin Endocrinol Metab.* (2018) 103:3439–48. 10.1210/jc.2018-00652 29982482

[8] Lee DH Joo MC. Change in bone mineral density in stroke patients with osteoporosis or osteopenia. *Int J Environ Res Public Health.* (2022) 19:8954. 10.3390/ijerph19158954 35897324 PMC9332617

[9] Carda S Cisari C Invernizzi M Bevilacqua M. Osteoporosis after stroke: a review of the causes and potential treatments. *Cerebrovasc Dis.* (2009) 28:191–200. 10.1159/000226578 19571530

[10] Jorgensen L Engstad T Jacobsen BK. Bone mineral density in acute stroke patients: low bone mineral density may predict first stroke in women. *Stroke.* (2001) 32:47–51. 10.1161/01.STR.32.1.47 11136913

[11] Myint PK Clark AB Kwok CS Loke YK Yeong JK Luben RN Bone mineral density and incidence of stroke: European prospective investigation into cancer-norfolk population-based study, systematic review, and meta-analysis. *Stroke.* (2014) 45:373–82. 10.1161/STROKEAHA.113.002999 24399373

[12] Nordstrom A Eriksson M Stegmayr B Gustafson Y Nordström P. Low bone mineral density is an independent risk factor for stroke and death. *Cerebrovasc Dis.* (2010) 29:130–6. 10.1159/000262308 19955736

[13] Lee SB Cho AH Butcher KS Kim TW Ryu SY Kim YI. Low bone mineral density is associated with poor clinical outcome in acute ischemic stroke. *Int J Stroke.* (2013) 8:68–72. 10.1111/j.1747-4949.2011.00714.x 22151871

[14] Fontalis A Kenanidis E Kotronias RA Papachristou A Anagnostis P Potoupnis M Current and emerging osteoporosis pharmacotherapy for women: state of the art therapies for preventing bone loss. *Expert Opin Pharmacother.* (2019) 20:1123–34. 10.1080/14656566.2019.1594772 30958709

[15] Schnitzer TJ Harvey RL Nack SH Supanwanid P Maskala-Streff L Roth E. Bone mineral density in patients with stroke: relationship with motor impairment and functional mobility. *Top Stroke Rehabil.* (2012) 19:436–43. 10.1310/tsr1905-436 22982831

[16] Borschmann K Pang MY Bernhardt J Iuliano-Burns S. Stepping towards prevention of bone loss after stroke: a systematic review of the skeletal effects of physical activity after stroke. *Int J Stroke.* (2012) 7:330–5. 10.1111/j.1747-4949.2011.00645.x 21967614

[17] Hamdy RC Moore SW Cancellaro VA Harvill LM. Long-term effects of strokes on bone mass. *Am J Phys Med Rehabil.* (1995) 74:351–6. 10.1097/00002060-199509000-00006 7576411

[18] Lee SH Park SY Jang MU Kim Y Lee J Kim C Association between osteoporosis and cognitive impairment during the acute and recovery phases of ischemic stroke. *Medicina.* (2020) 56:307. 10.3390/medicina56060307 32585806 PMC7353884

[19] Moayyeri A Alrawi YA Myint PK. The complex mutual connection between stroke and bone health. *Arch Biochem Biophys.* (2010) 503:153–9. 10.1016/j.abb.2010.06.023 20599661

[20] Lazoura O Groumas N Antoniadou E Papadaki PJ Papadimitriou A Thriskos P Bone mineral density alterations in upper and lower extremities 12 months after stroke measured by peripheral quantitative computed tomography and Dxa. *J Clin Densitom.* (2008) 11:511–7. 10.1016/j.jocd.2008.05.097 18639477

[21] Sato Y. Abnormal bone and calcium metabolism in patients after stroke. *Arch Phys Med Rehabil.* (2000) 81:117–21. 10.1053/apmr.2000.0810117 10638886

[22] Beaupre GS Lew HL. Bone-density changes after stroke. *Am J Phys Med Rehabil.* (2006) 85:464–72. 10.1097/01.phm.0000214275.69286.7a 16628156

[23] Liu M Tsuji T Higuchi Y Domen K Tsujiuchi K Chino N. Osteoporosis in hemiplegic stroke patients as studied with dual-energy X-ray absorptiometry. *Arch Phys Med Rehabil.* (1999) 80:1219–26. 10.1016/S0003-9993(99)90019-9 10527077

[24] Lee JE Jeon HR Park JK Han KD Kim JH Yoon J Sex differences in the association between stroke and bone mineral density in elderly Koreans: the Korean National Health and Nutrition Examination Survey, 2008-2010. *Maturitas.* (2017) 95:1–5. 10.1016/j.maturitas.2016.10.006 27889047

[25] Khan S Rahman MM Ataullah AH Rashid R. Vitamin D: the silent rescuer from ischemic stroke. *Ann Med Surg.* (2022) 78:103751. 10.1016/j.amsu.2022.103751 35620044 PMC9127262

[26] Poole KE Warburton EA Reeve J. Rapid long-term bone loss following stroke in a man with osteoporosis and atherosclerosis. *Osteoporos Int.* (2005) 16:302–5. 10.1007/s00198-004-1682-6 15197547

[27] Wang Q Zhu Z Liu Y Tu X He J. Relationship between serum vitamin D levels and inflammatory markers in acute stroke patients. *Brain Behav.* (2018) 8:e00885. 10.1002/brb3.885 29484258 PMC5822590

[28] Pirih F Lu J Ye F Bezouglaia O Atti E Ascenzi MG Adverse effects of hyperlipidemia on bone regeneration and strength. *J Bone Miner Res.* (2012) 27:309–18. 10.1002/jbmr.541 21987408 PMC3274629

[29] Tintut Y Morony S Demer LL. Hyperlipidemia promotes osteoclastic potential of bone marrow cells ex vivo. *Arterioscler Thromb Vasc Biol.* (2004) 24:e6–10. 10.1161/01.ATV.0000112023.62695.7f 14670933

[30] Graham LS Tintut Y Parhami F Kitchen CM Ivanov Y Tetradis S Bone density and hyperlipidemia: the T-lymphocyte connection. *J Bone Miner Res.* (2010) 25:2460–9. 10.1002/jbmr.148 20533376 PMC3179287

[31] Ngamruengphong S Leontiadis GI Radhi S Dentino A Nugent K. Proton pump inhibitors and risk of fracture: a systematic review and meta-analysis of observational studies. *Am J Gastroenterol.* (2011) 106:1209–18. quiz 19. 10.1038/ajg.2011.113 21483462

[32] Arj A Razavi Zade M Yavari M Akbari H Zamani B Asemi Z. Proton pump inhibitors use and change in bone mineral density. *Int J Rheum Dis.* (2016) 19:864–8. 10.1111/1756-185X.12866 27242025

[33] Yu EW Bauer SR Bain PA Bauer DC. Proton pump inhibitors and risk of fractures: a meta-analysis of 11 international studies. *Am J Med.* (2011) 124:519–26. 10.1016/j.amjmed.2011.01.007 21605729 PMC3101476

[34] Targownik LE Goertzen AL Luo Y Leslie WD. Long-term proton pump inhibitor use is not associated with changes in bone strength and structure. *Am J Gastroenterol.* (2017) 112:95–101. 10.1038/ajg.2016.481 27845341

[35] Kaye JA Jick H. Proton pump inhibitor use and risk of hip fractures in patients without major risk factors. *Pharmacotherapy.* (2008) 28:951–9. 10.1592/phco.28.8.951 18657011

[36] Igase M Kohara K Igase K Yamashita S Fujisawa M Katagi R. Prevalence and associated clinical factors of Gerd (gastro-esophageal reflux disease) in ischemic stroke patients. *Cerebrovas Dis.* (2013) 7. 10.4172/2155-9562.S8-004

[37] Niigaki M Adachi K Hirakawa K Furuta K Kinoshita Y. Association between metabolic syndrome and prevalence of gastroesophageal reflux disease in a health screening facility in Japan. *J Gastroenterol.* (2013) 48:463–72. 10.1007/s00535-012-0671-3 22976934

[38] Momosaki R Yasunaga H Matsui H Fushimi K Abo M. Proton pump inhibitors versus histamine-2 receptor antagonists and risk of pneumonia in patients with acute stroke. *J Stroke Cerebrovasc Dis.* (2016) 25:1035–40. 10.1016/j.jstrokecerebrovasdis.2016.01.018 26853142

[39] Lin SM Yang SH Liang CC Huang HK. Proton pump inhibitor use and the risk of osteoporosis and fracture in stroke patients: a population-based cohort study. *Osteoporos Int.* (2018) 29:153–62. 10.1007/s00198-017-4262-2 29032384

[40] Hackett ML Pickles K. Part I: frequency of depression after stroke: an updated systematic review and meta-analysis of observational studies. *Int J Stroke.* (2014) 9:1017–25. 10.1111/ijs.12357 25117911

[41] Cipriani A Furukawa TA Salanti G Chaimani A Atkinson LZ Ogawa Y Comparative efficacy and acceptability of 21 antidepressant drugs for the acute treatment of adults with major depressive disorder: a systematic review and network meta-analysis. *Lancet.* (2018) 391:1357–66. 10.1016/S0140-6736(17)32802-7 29477251 PMC5889788

[42] Jones JS Kimata R Almeida OP Hankey GJ. Risk of fractures in stroke patients treated with a selective serotonin reuptake inhibitor: a systematic review and meta-analysis. *Stroke.* (2021) 52:2802–8. 10.1161/STROKEAHA.120.032973 34167325

[43] Richter D Charles James J Ebert A Katsanos AH Mazul-Wach L Ruland Q Selective serotonin reuptake inhibitors for the prevention of post-stroke depression: a systematic review and meta-analysis.. *J Clin Med.* (2021) 10:5912. 10.3390/jcm10245912 34945207 PMC8704665

[44] Zhao D Liu J Wang M Zhang X Zhou M. Epidemiology of cardiovascular disease in China: current features and implications. *Nat Rev Cardiol.* (2019) 16:203–12. 10.1038/s41569-018-0119-4 30467329

[45] Peluso I Morabito G Urban L Zhang X Zhou M. Oxidative stress in atherosclerosis development: the central role of Ldl and oxidative burst. *Endocr Metab Immune Disord Drug Targets.* (2012) 12:351–60. 10.2174/187153012803832602 23061409

[46] Zhang T Jiang Y Zhang S Tie T Cheng Y Su X The association between homocysteine and ischemic stroke subtypes in Chinese: a meta-analysis. *Medicine.* (2020) 99:e19467. 10.1097/MD.0000000000019467 32195946 PMC7220264

[47] Hansson GK Hermansson A. The immune system in atherosclerosis. *Nat Immunol.* (2011) 12:204–12. 10.1038/ni.2001 21321594

[48] Moretti R Caruso P. The controversial role of homocysteine in neurology: from labs to clinical practice. *Int J Mol Sci.* (2019) 20:231. 10.3390/ijms20010231 30626145 PMC6337226

[49] Forstermann U Sessa WC. Nitric oxide synthases: regulation and function. *Eur Heart J.* (2012) 33:829–37. 37a-37d. 10.1093/eurheartj/ehr304 21890489 PMC3345541

[50] Ren JX Li C Yan XL Qu Y Yang Y Guo ZN. Crosstalk between oxidative stress and Ferroptosis/Oxytosis in ischemic stroke: possible targets and molecular mechanisms. *Oxid Med Cell Longev.* (2021) 2021:6643382. 10.1155/2021/6643382 34055196 PMC8133868

[51] Landmesser U Spiekermann S Preuss C Sorrentino S Fischer D Manes C Angiotensin Ii induces endothelial xanthine oxidase activation: role for endothelial dysfunction in patients with coronary disease. *Arterioscler Thromb Vasc Biol.* (2007) 27:943–8. 10.1161/01.ATV.0000258415.32883.bf 17234726

[52] Forstermann U Xia N Li H. Roles of vascular oxidative stress and nitric oxide in the pathogenesis of atherosclerosis. *Circ Res.* (2017) 120:713–35. 10.1161/CIRCRESAHA.116.309326 28209797

[53] Kattoor AJ Pothineni NV Palagiri D Mehta JL. Oxidative stress in atherosclerosis. *Curr Atheroscler Rep.* (2017) 19:42. 10.1007/s11883-017-0678-6 28921056

[54] Forstermann U. Nitric oxide and oxidative stress in vascular disease. *Pflugers Arch.* (2010) 459:923–39. 10.1007/s00424-010-0808-2 20306272

[55] Forstermann U. Oxidative stress in vascular disease: causes, defense mechanisms and potential therapies. *Nat Clin Pract Cardiovasc Med.* (2008) 5:338–49. 10.1038/ncpcardio1211 18461048

[56] Mauricio MD Guerra-Ojeda S Marchio P Valles SL Aldasoro M Escribano-Lopez I Nanoparticles in medicine: a focus on vascular oxidative stress. *Oxid Med Cell Longev.* (2018) 2018:6231482. 10.1155/2018/6231482 30356429 PMC6178176

[57] Schröder K Zhang M Benkhoff S Mieth A Pliquett R Kosowski J Nox4 is a protective reactive oxygen species generating vascular Nadph oxidase. *Circ Res.* (2012) 110:1217–25. 10.1161/CIRCRESAHA.112.267054 22456182

[58] Maalouf RM Eid AA Gorin YC Block K Escobar GP Bailey S Nox4-derived reactive oxygen species mediate cardiomyocyte injury in early type 1 diabetes. *Am J Physiol Cell Physiol.* (2012) 302:C597–604. 10.1152/ajpcell.00331.2011 22031600 PMC3814247

[59] Marchio P Guerra-Ojeda S Vila JM Aldasoro M Victor VM Mauricio MD. Targeting early atherosclerosis: a focus on oxidative stress and inflammation. *Oxid Med Cell Longev.* (2019) 2019:8563845. 10.1155/2019/8563845 31354915 PMC6636482

[60] Domazetovic V Marcucci G Iantomasi T Brandi ML Vincenzini MT. Oxidative stress in bone remodeling: role of antioxidants. *Clin Cases Miner Bone Metab.* (2017) 14:209–16. 10.11138/ccmbm/2017.14.1.209 29263736 PMC5726212

[61] Agidigbi TS Kim C. Reactive oxygen species in osteoclast differentiation and possible pharmaceutical targets of ros-mediated osteoclast diseases. *Int J Mol Sci.* (2019) 20:3576. 10.3390/ijms20143576 31336616 PMC6678498

[62] Zou DB Mou Z Wu W Liu H. Trim33 protects osteoblasts from oxidative stress-induced apoptosis in osteoporosis by inhibiting Foxo3a ubiquitylation and degradation. *Aging Cell.* (2021) 20:e13367. 10.1111/acel.13367 34101965 PMC8282270

[63] Shang N Bhullar KS Hubbard BP Wu J. Tripeptide Irw initiates differentiation in osteoblasts differentiation via the Runx2 pathway. *Biochim Biophys Acta Gen Subj.* (2019) 1863:1138–46. 10.1016/j.bbagen.2019.04.007 30980895

[64] Geng Q Gao H Yang R Guo K Miao D. Pyrroloquinoline quinone prevents estrogen deficiency-induced osteoporosis by inhibiting oxidative stress and osteocyte senescence. *Int J Biol Sci.* (2019) 15:58–68. 10.7150/ijbs.25783 30662347 PMC6329928

[65] Baek KH Oh KW Lee WY Lee SS Kim MK Kwon HS Association of oxidative stress with postmenopausal osteoporosis and the effects of hydrogen peroxide on osteoclast formation in human bone marrow cell cultures. *Calcif Tissue Int.* (2010) 87:226–35. 10.1007/s00223-010-9393-9 20614110

[66] Kim EN Kim GR Yu JS Kim K Jeong G. Inhibitory effect of (2R)-4-(4-hydroxyphenyl)-2-butanol 2-O-beta-d-apiofuranosyl-(1–>6)-beta-d-glucopyranoside on Rankl-induced osteoclast differentiation and ros generation in macrophages. *Int J Mol Sci.* (2020) 22:222. 10.3390/ijms22010222 33379346 PMC7795186

[67] Li X Xu J Dai B Wang X Guo Q Qin L. Targeting autophagy in osteoporosis: from pathophysiology to potential therapy. *Ageing Res Rev.* (2020) 62:101098. 10.1016/j.arr.2020.101098 32535273

[68] Gong W Liu M Zhang Q Zhang Q Wang Y Zhao Q Orcinol glucoside improves senile osteoporosis through attenuating oxidative stress and autophagy of osteoclast via activating Nrf2/Keap1 and mtor signaling pathway. *Oxid Med Cell Longev.* (2022) 2022:5410377. 10.1155/2022/5410377 35585885 PMC9110208

[69] Greenberg JA Roth EJ Wuermser LA Almagor O Schnitzer TJ. Osteoporosis treatment for patients with stroke. *Top Stroke Rehabil.* (2007) 14:62–7. 10.1310/tsr1402-62 17517576

[70] Howe TE Shea B Dawson LJ Downie F Murray A Ross C Exercise for preventing and treating osteoporosis in postmenopausal women. *Cochrane Database Syst Rev.* (2011) 6:Cd000333. 10.1002/14651858.CD000333.pub2 21735380 PMC12744941

[71] Pinheiro MB Oliveira J Bauman A Fairhall N Kwok W Sherrington C. Evidence on physical activity and osteoporosis prevention for people aged 65+ years: a systematic review to inform the who guidelines on physical activity and sedentary behaviour. *Int J Behav Nutr Phys Act.* (2020) 17:150. 10.1186/s12966-020-01040-4 33239014 PMC7690138

[72] Sherrington C Fairhall NJ Wallbank GK Tiedemann A Michaleff ZA Howard K Exercise for preventing falls in older people living in the community. *Cochrane Database Syst Rev.* (2019) 1:Cd012424. 10.1002/14651858.CD012424.pub2 30703272 PMC6360922

[73] Wimalawansa SJ Razzaque MS Al-Daghri NM. Calcium and vitamin D in human health: hype or real? *J Steroid Biochem Mol Biol.* (2018) 180:4–14. 10.1016/j.jsbmb.2017.12.009 29258769

[74] Vuolo L Di Somma C Faggiano A Colao A. Vitamin D and cancer. *Front Endocrinol.* (2012) 3:58. 10.3389/fendo.2012.00058 22649423 PMC3355893

[75] Kearns MD Alvarez JA Seidel N Tangpricha V. Impact of vitamin D on infectious disease. *Am J Med Sci.* (2015) 349:245–62. 10.1097/MAJ.0000000000000360 25334038 PMC4346469

[76] Yang CY Leung PS Adamopoulos IE Gershwin ME. The implication of vitamin D and autoimmunity: a comprehensive review. *Clin Rev Allergy Immunol.* (2013) 45:217–26. 10.1007/s12016-013-8361-3 23359064 PMC6047889

[77] Roider E Ruzicka T Schauber J. Vitamin d, the cutaneous barrier, antimicrobial peptides and allergies: is there a link? *Allergy Asthma Immunol Res.* (2013) 5:119–28. 10.4168/aair.2013.5.3.119 23638309 PMC3636445

[78] Rejnmark L. Effects of vitamin d on muscle function and performance: a review of evidence from randomized controlled trials. *Ther Adv Chronic Dis.* (2011) 2:25–37. 10.1177/2040622310381934 23251739 PMC3513873

[79] Targher G Pichiri I Lippi G. Vitamin D, thrombosis, and hemostasis: more than skin deep. *Semin Thromb Hemost.* (2012) 38:114–24. 10.1055/s-0031-1300957 22314609

[80] Kim D. The role of vitamin D in thyroid diseases. *Int J Mol Sci.* (2017) 18:1949. 10.3390/ijms18091949 28895880 PMC5618598

[81] Pittas AG Laskowski U Kos L Saltzman E. Role of vitamin D in adults requiring nutrition support. *Jpen J Parenter Enteral Nutr.* (2010) 34:70–8. 10.1177/0148607109349061 19875748 PMC2857395

[82] Akimbekov NS Ortoski RA Razzaque MS. Effects of sunlight exposure and vitamin D supplementation on Hiv patients. *J Steroid Biochem Mol Biol.* (2020) 200:105664. 10.1016/j.jsbmb.2020.105664 32229174

[83] Eastell R Rosen CJ Black DM Cheung AM Murad MH Shoback D. Pharmacological management of osteoporosis in postmenopausal women: an endocrine society* clinical practice guideline. *J Clin Endocrinol Metab.* (2019) 104:1595–622. 10.1210/jc.2019-00221 30907953

[84] Marsden J Gibson LM Lightbody CE Sharma AK Siddiqi M Watkins C. Can early onset bone loss be effectively managed in post-stroke patients? An integrative review of the evidence. *Age Ageing.* (2008) 37:142–50. 10.1093/ageing/afm198 18349011

[85] Body JJ Bergmann P Boonen S Devogelaer J Gielen E Goemaere S Extraskeletal benefits and risks of calcium, vitamin D and anti-osteoporosis medications. *Pain Joints Spine.* (2013) 23:S1–23. 10.1007/s00198-011-1891-8 22311111 PMC3273686

[86] Makariou SE Michel P Tzoufi MS Challa A Milionis HJ. Vitamin D and stroke: promise for prevention and better outcome. *Curr Vasc Pharmacol.* (2014) 12:117–24. 10.2174/15701611113119990119 22724468

[87] Poole KE Reeve J Warburton EA. Falls, fractures, and osteoporosis after stroke: time to think about protection? *Stroke.* (2002) 33:1432–6. 10.1161/01.STR.0000014510.48897.7D 11988628

[88] Martins D Wolf M Pan D Zadshir A Tareen N Thadhani R Prevalence of cardiovascular risk factors and the serum levels of 25-hydroxyvitamin D in the United States: data from the Third National Health and Nutrition Examination Survey. *Arch Intern Med.* (2007) 167:1159–65. 10.1001/archinte.167.11.1159 17563024

[89] Stojanovic OI Lazovic M Lazovic M Vuceljic M. Association between atherosclerosis and osteoporosis, the role of vitamin D. *Arch Med Sci.* (2011) 7:179–88. 10.5114/aoms.2011.22066 22291755 PMC3258717

[90] Uluduz D Adil MM Rahim B Gilani WI Rahman HA Gilani SI Stroke. Retraction to: vitamin D deficiency and risk of hip fractures among disabled elderly stroke patients. *Stroke.* (2019) 50:e247. 10.1161/STR.0000000000000201 32812485

[91] Uluduz D Adil MM Rahim B Gilani WI Rahman HA Gilani SI Vitamin D deficiency and osteoporosis in stroke survivors: an analysis of National Health and Nutritional Examination Survey (Nhanes). *J Vasc Interv Neurol.* (2014) 7:23–8. PMC4051901 24920985

[92] Yao P Bennett D Mafham M Lin X Chen Z Armitage J Vitamin D and calcium for the prevention of fracture: a systematic review and meta-analysis. *Jama Netw Open.* (2019) 2:e1917789. 10.1001/jamanetworkopen.2019.17789 31860103 PMC6991219

[93] Dolan E Varley I Ackerman KE Pereira RMR Elliott-Sale KJ Sale C. The bone metabolic response to exercise and nutrition. *Exerc Sport Sci Rev.* (2020) 48:49–58. 10.1249/JES.0000000000000215 31913188

[94] Han L Li SG Zhai HW Guo PF Chen W. Effects of weight training time on bone mineral density of patients with secondary osteoporosis after hemiplegia. *Exp Ther Med.* (2017) 13:961–5. 10.3892/etm.2017.4078 28450926 PMC5403320

[95] Sallehuddin H Ong T Md Said S Ahmad Tarmizi NA Loh SP Lim WC Non-pharmacological interventions for bone health after stroke: a systematic review. *PLoS One.* (2022) 172:e0263935. 10.1371/journal.pone.0263935 35196338 PMC8865685

[96] An T Hao J Sun S Li R Yang M Cheng G Efficacy of statins for osteoporosis: a systematic review and meta-analysis. *Osteoporos Int.* (2017) 28:47–57. 10.1007/s00198-016-3844-8 27888285

[97] Jin S Jiang J Bai P Zhang M Tong X Wang H Statin use and risk of fracture: a meta-analysis. *Int J Clin Exp Med.* (2015) 8:8269–75. 26221409 PMC4509354

[98] Wang Z Li Y Zhou F Piao Z Hao J. Effects of statins on bone mineral density and fracture risk: a prisma-compliant systematic review and meta-analysis. *Medicine*, (2016) 95:e3042. 10.1097/MD.0000000000003042 27258488 PMC4900696

[99] Sato Y Iwamoto J Kanoko T Satoh K. Risedronate therapy for prevention of hip fracture after stroke in elderly women. *Neurology.* (2005) 64:811–6. 10.1212/01.WNL.0000152871.65027.76 15753414

[100] Poole KE Vedi S Debiram I Rose C Power J Loveridge N Bone structure and remodelling in stroke patients: early effects of zoledronate. *Bone.* (2009) 44:629–33. 10.1016/j.bone.2008.11.017 19121416 PMC2724102

[101] Sato Y Kuno H Kaji M Etoh K Oizumi K. Influence of immobilization upon calcium metabolism in the week following hemiplegic stroke. *J Neurol Sci.* (2000) 175:135–9. 10.1016/s0022-510x(00)00298-7 10831774

